# Wie wirken mediale Altersbilder auf ältere Menschen? – Ergebnisse einer Rezeptionsstudie

**DOI:** 10.1007/s00391-020-01745-y

**Published:** 2020-06-30

**Authors:** Julian Wangler, Michael Jansky

**Affiliations:** Zentrum für Allgemeinmedizin und Geriatrie, Universitätsmedizin Mainz, Am Pulverturm 13, 55131 Mainz, Deutschland

**Keywords:** Altersbilder, Altersdarstellungen, Framing, Frame, Selbstbild, Fremdbild, Wirkungen, Rezeption, Old age images, Old age representations, Framing, Frame, Self-image, Foreign image, Effects, Reception

## Abstract

**Hintergrund:**

Seit Längerem wird über die Bedeutung medienvermittelter Altersbilder diskutiert. Dennoch gibt es bislang kaum empirische Anhaltspunkte, inwiefern solche durch Medien kommunizierte (Re)Präsentationen des höheren Lebensalters einstellungsverändernde Effekte bei Rezipienten auslösen können. Im Zentrum der Studie steht die Frage, wie sich (prototypische) mediale Altersdarstellungen auf Einstellungen zum Alter (Altersfremdbild) und zum persönlichen Älterwerden (Altersselbstbild) auswirken.

**Material und Methoden:**

Im Zuge einer Inhaltsanalyse wurden 3 verbreitete Altersdarstellungen in den Nachrichtenmagazinen *Spiegel, Stern* und *Focus* bestimmt und, darauf aufbauend, prototypisches Stimulusmaterial selektiert („frames“). Anschließend wurden 910 Teilnehmende zwischen 60 und 94 Jahren, die in 3 Experimentalgruppen eingeteilt wurden, im Zuge einer quasiexperimentellen Befragung mit jeweils einem Alters-Frame konfrontiert. Im Mittelpunkt stand ein Vorher-nachher-Vergleich von Indikatoren zu Altersfremdbild und -selbstbild.

**Ergebnisse:**

Im Vorher-nachher-Vergleich verändern sich die Indikatoren zum Altersfremdbild stärker als das Altersselbstbild. Zudem klaffen Selbst- und Fremdbildindikatoren auseinander. Die Vorlage des negativen Alters-Frame führt zu einer Verbesserung des Selbstbilds, während sich das Fremdbild deutlich verschlechtert. Umgekehrt verbessert sich nach Vorlage des positiven Frame das Fremdbild stark, während das Selbstbild Einbußen erleidet.

**Diskussion:**

Mediale Altersdarstellungen scheinen sich nicht in der Weise auf das Altersfremdbild und -selbstbild älterer Personen auswirken, wie man auf Basis ihrer inhaltlichen Beschaffenheit vermuten könnte. Hingegen scheinen sie von Rezipienten für soziale Vergleiche genutzt zu werden. So scheinen negative Effekte z. B. dann einzutreten, wenn Mediendarstellungen älteren Rezipienten durch Präsentation (über)positiv inszenierter „best ager“ das eigene Alter(n) bewusst machen. Im Lichte dieser Ergebnisse lässt sich folglich die Theorie sozialer Vergleichsprozesse heranziehen, derzufolge Medien Rezipienten Vergleichsmaßstäbe an die Hand geben. Angesichts solcher Befunde sollten gängige Annahmen zur Wirkung von Altersbildern überdacht werden. Auch die Interaktion von Altersfremdbild und -selbstbild sollte verstärkt beachtet werden.

**Zusatzmaterial online:**

Zusätzliche Informationen sind in der Online-Version dieses Artikels (10.1007/s00391-020-01745-y) enthalten.

## Abstract (en)

**Background:**

The importance of media-mediated images of old age and ageing has been discussed for a long time. Nevertheless, there is sparse empirical evidence on the effects media (re)presentations of old age can actually have on recipients. This study explored the question of how (prototypical) media representations of age affect attitudes towards old age (foreign image of age) and personal aging (self-image of age).

**Material and methods:**

In the course of a content analysis, three common age representations were identified in the news magazines *Spiegel*, *Stern* and *Focus*. Based on this, prototypical stimulating material was selected (frames). Subsequently, 910 participants between the ages of 60 and 94 years were divided into 3 experimental groups. Over the course of a quasi-experimental survey each participant was confronted with an age frame. The focus was on a before-after comparison of indicators relating to the foreign and self-images of age.

**Results:**

The before-after comparison showed that the indicators of the external perception of age changed more than the self-image. In addition, foreign and self-image indicators tended to diverge. The presentation of the negative frame led to an improvement of the self-image while the external image significantly deteriorated. Conversely, after the positive frame was presented, the external image significantly improved while the self-image suffered.

**Conclusion:**

Media depictions of age do not seem to have an impact on older people’s foreign and self-images as could be assumed based merely on the content. Moreover, the recipients seemed to use them for social comparisons. As a result, negative effects appeared to occur when media representations made older recipients aware of their own age by presenting positively staged best agers. In the light of these results, the theory of social comparison processes can be used, stating that media provide recipients with comparative standards. In view of these findings, common assumptions about the effects of old age images should be reconsidered. More attention should be paid to the interaction between foreign and self-images.

Massenmedial verbreitete Altersbilder haben für gesellschaftliche Vorstellungen von Alter und Älterwerden große Bedeutung. Bislang fehlen empirische Befunde, welche Wirkungen medienvermittelte (Re)Präsentationen vom höheren Lebensalter auf Rezipienten haben können. Im Zuge der vorliegenden Studie wurden 910 ältere Probanden mit prototypischen Altersdarstellungen aus der Nachrichtenberichterstattung konfrontiert. Dabei wurden nicht nur Reaktionen, sondern auch eine mögliche Beeinflussung von Altersfremdbild und -selbstbild erfasst.

## Hintergrund

Obwohl Alter ein fortschreitender Prozess und damit ein relatives Merkmal ist, haben viele Menschen intuitiv bestimmte Vorstellungen, anhand welcher Merkmale ältere Menschen identifiziert werden können [22, 24]. Die dahinter stehenden Altersbilder können als Stereotype wirksam sein und individuelles Verhalten beeinflussen [7, 11, 21, 27]. Für die Frage, welche gesamtgesellschaftlichen Altersbilder geprägt werden, spielen heute Massenmedien eine entscheidende Rolle [9]. Medien selektieren und heben bestimmte Aspekte des Alters hervor [3, 15]. Auf diese Weise entstehen kohärente Darstellungs- und Deutungsmuster, die polarisierende Optiken und Eigenschaftszuschreibungen in Bezug auf die abschließende Lebensphase eröffnen [5].

Bislang erschöpfte sich die Erforschung medialer Altersbilder weitgehend darin, Medienerzeugnisse inhaltsanalytisch zu untersuchen und auf Wirkungen zu schließen [3, 10, 22]. Entsprechend liegen kaum Befunde vor, welche (einstellungsverändernden) Effekte medienvermittelte (Re)Präsentationen des Alters tatsächlich auslösen [9]. Denkbar sind unterschiedliche Wirkungspotenziale, die sich an einer Reihe von Theorien aus der sozialpsychologischen Einstellungsforschung verdeutlichen lassen. Bei ihnen steht die gegenseitige Beeinflussung von Altersfremdbildern und -selbstbildern im Mittelpunkt [1, 8]. Während das Altersfremdbild als eine Art „Außenbetrachtung“ auf die abschließende Lebensphase gelten kann, das vermeintlich typische Eigenschaften sowie Verhaltens- und Rollenerwartungen bereithält, beinhaltet das Altersselbstbild v. a. das subjektive Alterserleben und eine Projektion des künftigen älterwerdenden Selbst. Im Fokus dieser Theorien stehen besonders ältere Menschen, weil davon auszugehen ist, dass sie über ein gleichermaßen entwickeltes Fremd- wie Selbstbild des Alters verfügen [24].

Im Verständnis der *Terror-Management-Theorie* wird die Konfrontation mit einem Altersbild, das Gebrechlichkeit, Hilflosigkeit und Sterblichkeit bereithält, als bedrohlich empfunden [14]. Die *Internalisierungshypothese *unterstellt, dass ältere Menschen dazu neigen, negative Altersstereotype zu verinnerlichen [19], was Selbstkonzept und Resilienz schädigen kann [7, 18]. Die Internalisierungshypothese bewegt sich nah am „Ageism“-Postulat, bei dem ausgehend von einer öffentlichen Stereotypisierung älterer Menschen eine höhere Prävalenz für seelische und physische Erkrankungen im Alter sowie erhöhte Kosten für das Gesundheitssystem angenommen werden [12]. Umgekehrt kann angenommen werden, dass positive Altersbilder das Selbstbild unterstützen [20]. Vor dem Hintergrund der *Resilienztheorie* ist indes von einer gewissen Barriere bei der Übertragung von negativen Altersfremdbildern auf das Selbstbild auszugehen, da ältere Menschen bestrebt sind, ihr Selbstkonzept zu schützen [28].

Die *Vergleichshypothese *erachtet Altersbilder als Referenzpunkte für soziale Vergleiche. Statt negative Informationen in ihr Selbstbild zu integrieren, wird ein selbstwertdienlicher Abwärtsvergleich mit dem negativen Altersstereotyp vollzogen [11, 13]. Auf diese Weise kann es zu einer Instrumentalisierung negativer Altersbilder zwecks Verbesserung des Selbstwerts kommen [9]. Die *Verstärkerhypothese* geht davon aus, dass die Art und Weise, wie Altersfremdbilder empfangen und erlebt werden, stark von den Voraussetzungen des Selbstbilds abhängt [2, 5, 21, 23]. So muss als Bedingung für eine Widerstandsfähigkeit gegen negative Altersbilder ein intaktes Selbstbild gegeben sein. Dadurch nehmen ältere Menschen v. a. positive Aspekte des auf sie einströmenden Fremdbilds wahr und sind mitunter auch in der Lage, selbstwertdienliche Aufwärtsvergleiche vorzunehmen.

Im Zentrum der Studie steht die folgende Forschungsfrage: Wie wirken sich (prototypische) mediale Altersdarstellungen auf Einstellungen zum Alter (Altersfremdbild) und zum persönlichen Älterwerden (Altersselbstbild) aus? Basierend auf den theoretischen Vorannahmen wurde die folgende Hypothese bezüglich der anzunehmenden Beeinflussung des Altersselbstbilds und -fremdbilds formuliert: Die Veränderung des Altersselbstbilds und -fremdbilds entspricht der Darstellungs- bzw. Bewertungstendenz von Alter bzw. älteren Menschen im Frame-Stimulus.

Als forschungsleitendes Konzept wird der Framing-Ansatz verwendet [4]. Altersbilder werden als spezifische Darstellungs- und Deutungsmuster (Frames) operationalisiert, in denen bestimmte Aspekte des höheren Lebensalters betont und dadurch eine spezifische „Lesart“ vorgegeben wird. Als Zielgruppe wurden ältere Menschen ausgewählt, da davon auszugehen ist, dass sie mediale Altersbilder in besonderer Weise wahrnehmen und verarbeiten.

## Methodik

Die Studie basiert auf der Dissertationsarbeit des Erstautors aus dem Jahr 2013, in der sie konzeptionell erprobt wurde [29]. Da es nach wie vor an Arbeiten zur Wirksamkeit von medienkommunizierten Altersbildern fehlt, wurde die Studie aktualisiert und in größerem Maßstab wiederholt, um zu prüfen, inwieweit sich die damaligen Resultate bestätigen lassen.

Im Mittelpunkt steht eine quasiexperimentelle schriftliche Befragung^1^, mit deren Hilfe analysiert werden soll, wie ältere Menschen auf prägnante Alters-Frames reagieren. Hierzu wurde in der Mitte des Fragebogens ein prototypischer Alters-Frame platziert. Gemessen wird u. a., wie der Stimulus sich auf zentrale Indikatoren zum Altersfremdbild und -selbstbild auswirkt^2^ (Vorher-nachher-Vergleich von 3 im Anschluss an die Rezeption wiederholt ermittelten Items).

Die vollständig anonymisierte Befragung wurde zwischen Februar und November 2019 durchgeführt und war durch eine inhaltsanalytische Vorstudie abgestützt.

### Inhaltsanalytische Vorstudie

Im Vorfeld wurde eine qualitative Inhaltsanalyse durchgeführt, mit der verbreitete Darstellungsmuster identifiziert werden sollten. Zudem ging es darum, aussagekräftiges Stimulusmaterial für das Fragebogenexperiment zu gewinnen.

Mithilfe der Datenbank *LexisNexis* wurde die Berichterstattung in den Nachrichtenmagazinen *Spiegel, Stern* und *Focus* betrachtet. Zugriffskriterien waren der Zeitraum 01.01.1999 bis 31.12.2019, ein Artikelumfang ab 500 Wörter und ein inhaltliches Kriterium, demzufolge Alter bestimmendes Thema des Beitrags sein muss.

Die Inhaltsanalyse [16] mündete in ein Kategoriensystem (Zusatzmaterial online: Anhang 1), das die Frame-definierenden Indikatoren enthält, darunter: Thema, Aktivität/Passivität älterer Menschen, zahlenmäßiges Auftreten älterer Menschen, Umfeld älterer Menschen, Geschlecht älterer Menschen, Schichtzugehörigkeit älterer Menschen, Beziehung Alt-Jung, Beziehung Alt-Alt, Rolle älterer Menschen. Im Zuge einer zweiten Materialsichtung wurden die Frames auf Grundlage der Indikatoren abgeleitet.

Anschließend wurde das Stimulusmaterial selektiert. Hier war für den jeweiligen Frame derjenige Artikel erste Wahl, in dem wichtige Indikatoren am idealtypischsten ausgeprägt sind und keine konkurrierenden Frames auftreten. Anschließend wurde der Beitrag nach folgenden Kriterien angepasst:

- maximal 400 Wörter,
- Erhaltung für den Text wichtiger persönlicher Beispiele,
- weitgehende Kürzung ohne Veränderung der Kernaussagen und Argumentation.

In der ursprünglichen Studie erfolgte eine Testung des Stimulusmaterials im Rahmen fokussierter Interviews. Aufgrund dieser Erprobung wurde nun auf einen solchen Zwischenschritt verzichtet.

### Rekrutierung

Die Versuchspersonen wurden in Südhessen und Rheinland-Pfalz rekrutiert, wobei es sich jeweils zur Hälfte um Einzelpersonen handelt sowie um Gruppen älterer Menschen, die im Zuge eines Besuchs örtlicher Seniorengruppen und kommunaler Seniorentreffs gewonnen werden konnten. Im Vorfeld erfolgte stets eine telefonische oder E‑Mail-Anfrage, in der eine allgemeine Erlaubnis eingeholt wurde, den entsprechenden Seniorentreff zu besuchen, die Mitglieder für die Studie zu gewinnen und diese durchführen zu dürfen. Versuchsleiter war der Verfasser. Die Teilnehmer wurden über die Studie sowie über die Absicht einer Veröffentlichung anonymisierter Daten aufgeklärt und um Einwilligung gebeten. Die Versuchspersonen erhielten die Anweisung, beim Ausfüllen weder vor- noch zurückzublättern. Es wurde besonderer Wert darauf gelegt, die Befragten erst nach Beendigung und Wiedereinsammlung der Befragung über das zentrale Erkenntnisinteresse zu informieren, um ein strategisches Antwortverhalten zu verhindern.

Wurde in der ursprünglichen Arbeit mit einer kleinen und willkürlichen Stichprobe operiert, sollten nun ca. 1000 Personen ab 60 Jahre befragt und zudem der Versuch einer systematischeren Rekrutierung unternommen werden^3^. Eine Power-Analyse hat nicht stattgefunden. Im Rahmen der Befragung existieren 3 gleich große und im Hinblick auf Alter und Geschlecht gleichartig aufgebaute Experimentalgruppen, die jeweils einen Alters-Frame erhielten.

### Stichprobe

Insgesamt 910 Befragungsteilnehmer haben den Fragebogen vollständig ausgefüllt (43 unvollständig und nicht in die Auswertung einbezogen). Die Befragten waren zwischen 60 und 94 Jahre alt, während der Altersdurchschnitt bei 72 Jahren liegt (Median: 71, s: 7,6). 47 % sind männlich, 53 % weiblich. Weitere Merkmale der Gesamtstichprobe sind:

- Staatsangehörigkeit: 94 % deutsch, 6 % andere,
- Wohnsituation: 79 % eigene Wohnung, 12 % Seniorenresidenz/Pflegeheim, 9 % Sonstiges,
- Personen im Haushalt: 25 % allein lebend, 69 % zwei Personen, 6 % mehr als 2 Personen,
- höchster Bildungsabschluss: 13 % Volks‑/Hauptschule, 18 % mittlere Reife/Realschule, 8 % (Fach)Abitur, 28 % (Fach)Hochschulabschluss, 33 % Berufsausbildung.

Die Zusammensetzung der 3 Experimentalgruppen zeigt Tab. 1.

**Table 1:** 

	Gruppe 1 (*n* = 309; in %)	Gruppe 2 (*n* = 303; in %)	Gruppe 3 (*n* = 298; in %)
60–70 Jahre	46,0	48,5	45,3
71–85 Jahre	43,4	42,9	47,7
86+ Jahre	10,6	8,6	7,0

### Erhebungsinstrument

Das Erhebungsinstrument (Zusatzmaterial online: Anhang 2) wurde 2012 auf Grundlage einer Literaturrecherche sowie einer ergänzenden qualitativen Vorstudie erstellt, bei denen das Stimulusmaterial im Zuge von 12 fokussierten Interviews mit älteren Personen vorgetestet wurde. Folglich enthält der Fragebogen induktiv und deduktiv abgeleitete Bestandteile.

**Figure 1:**
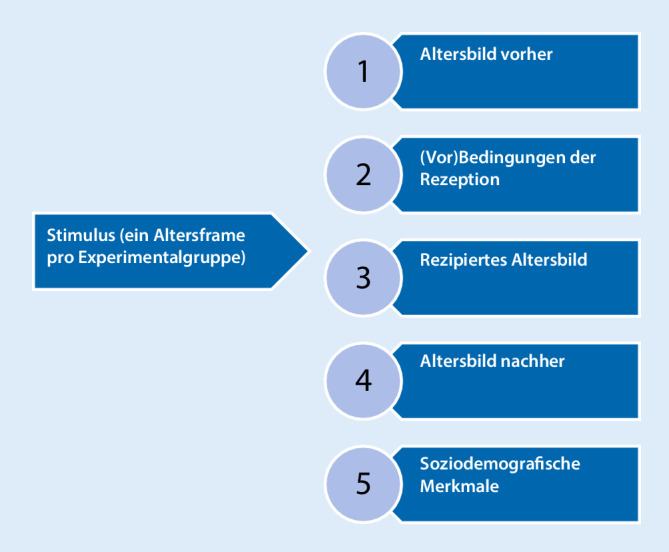

Eine Übersicht über die inhaltlichen Komponenten der Befragung bietet Abb. 1. Im Mittelpunkt der Untersuchung stehen ausgewählte Indikatoren zum Altersselbstbild und -fremdbild (Komponenten 1 und 4), die in der Untersuchung die abhängige Variable repräsentieren und jeweils vor und nach der Konfrontation mit dem Stimulus (unabhängige Variable) abgefragt werden. Der Stimulus beinhaltet einen prototypischen Alters-Frame. Des Weiteren wird das rezipierte Altersbild (Komponente 3) ermittelt, das sich auf Wahrnehmung und Beurteilung der Altersdarstellung bezieht. Nicht zuletzt werden als moderierende Variablen ausgewählte (Vor)Bedingungen der Rezeption (Komponente 2) sowie soziodemografische Merkmale (Komponente 5) einbezogen.

Für sämtliche verwendeten Skalen (Zusatzmaterial online: Anhang 2) wurde die interne Konsistenz berechnet. Diese lässt sich für die zu berichtenden Skalen als gut bezeichnen:

- Skala: emotionale Reaktion (Tab. 3); Cronbachs α = 0,891,
- Skala: Texteinschätzung (Tab. 4); Cronbachs α = 0,832,
- Skala: Verhältnis zwischen Alt und Jung (Tab. 5); Cronbachs α = 0,855,
- Skala: Altersselbstbild vorher (Tab. 6); Cronbachs α = 0,803,
- Skala: Altersfremdbild vorher (Tab. 6); Cronbachs α = 0,843,
- Skala: Altersselbstbild nachher (Tab. 6); Cronbachs α = 0,901,
- Skala: Altersfremdbild nachher (Tab. 6); Cronbachs α = 0,824.

### Datenanalyse

Neben der deskriptiven Analyse kam zur Feststellung von signifikanten Unterschieden innerhalb der Experimentalgruppen ein *t*-Test für abhängige Stichproben (Messwiederholung) zum Einsatz. Überprüft wurde eine Mittelwertdifferenz auf dem Niveau *p* < 0,001.

## Ergebnisse

### Frame-Identifikation

Es konnten 3 wiederkehrende und polarisierende Alters-Frames identifiziert werden, die sich durch einen Bezug zu den Themen demografischer Wandel, Generationenverhältnis und Sozialstaat auszeichnen (Tab. 2):

- Frame 1 („Alter als Niedergang“) modelliert Alter als umfassendes Problem und Leidensschicksal. Hierzu wird ein Lebensabschnitt großer Hilflosigkeit und Fremdbestimmung illustriert, v. a. am Beispiel von in Pflegeheimen untergebrachten älteren Menschen. Diese können nicht gegen Unrecht, das ihnen widerfährt, angehen, sondern sind vielfach Ausgelieferte entwürdigender Alltagssituationen. Eng damit verknüpft ist die Darstellung von ausgeprägter Einsamkeit im Alter.
- Frame 2 („Alter als [Über]Macht“) inszeniert Alter als Machtfaktor, der das Gemeinwesen politisch, ökonomisch und institutionell neu ordnet. Senioren treten als organisierte politische Gruppe auf und vertreten ihre Interessen zusehends offensiv. Insofern lassen sich Ältere als dominante Einfluss- und Wirkgröße begreifen, der in Politik und Alltag aufgrund der schieren demografischen Entwicklung immer mehr Macht zufällt. Mit Ansprüchen an mehr Wohlstand bedrohen sie dabei die jüngere Generation.
- Frame 3 („Alter als neuer Aufbruch“) schreibt Alter ein äußerst positives Image zu. Es erscheint als späte Freiheit mit glücklichen, erfüllten und wohlhabenden Senioren. Im Vordergrund steht, wie sich die Werbe‑, Elektronik- oder Gesundheitsindustrie auf vermögende und selbstbestimmte „best ager“ umorientiert. Ebenfalls werden die geistige Fitness, Lebensfreude und physische Vitalität älterer Menschen gezeigt. Indem die „jungen Alten“ das Konsumverhalten und den Lebensstil ihrer Kinder und Enkelkinder imitieren, heben sie traditionelle Altersvorstellungen auf.

**Table 2:** 

Frame	1: Alter als Niedergang	2: Alter als (Über)Macht	3: Alter als neuer Aufbruch
Thematischer Kontext	Pflege, Sozialpolitik, Krankheit/Sterben, Euthanasie	Renten- und Sozialpolitik, demografischer Wandel, Alters- und Seniorenlobbys, politische Parteien, Generationengerechtigkeit bzw. -konflikte	Gesundheit und Fitness, Konsum/Lifestyle, Arbeitsmarkt, Elektronik/Technik/Medien, freiwilliges Engagement, (Weiter)Bildung, Familie und Partnerschaft
Rolle älterer Menschen	Passiv	Aktiv	Aktiv
Verhältnis Alt zu Jung	Abhängigkeitsverhältnis	Spannungsreiches oder konfliktäres Verhältnis	Harmonisches bis neutrales Verhältnis
Bewertungstendenz im Hinblick auf Alter	Negativ	Negativ	Positiv

Die im Anhang 2 des Zusatzmaterial online befindlichen, entsprechend angepassten Stimulustexte 1 bis 3 beziehen sich auf die dargestellten Alters-Frames. Als Stimulustexte ausgewählt wurden folgende Artikel:

- Frame 1: Wedemeyer G (2002) Wo auch die Würde stirbt. *Stern*, Nr. 31, S. 46–52,
- Frame 2: Reich F (2009) Die graue Macht. *Stern*, Nr. 28, S. 58–66,
- Frame 3: Gerbert F (2007) Golden oldies. Generation happy end. *Focus*, Nr. 51, S. 108–113.

### Befragungsergebnisse

Blickt man auf die emotionalen Reaktionen im Anschluss an die Rezeption, zeigt sich, dass Frame 1 bei den Probanden überwiegend negative Gefühle auslöst (Tab. 3). Demgegenüber weckt der dritte Frame überwiegend positive Gefühle. Zugleich gibt ein Drittel an, dass diese Art der Altersdarstellung Schuldgefühle aufkommen lasse.

**Table 3:** 

Der Text …	1: Alter als Niedergang (*n* = 309; in %)	2: Alter als (Über)Macht (*n* = 303; in %)	3: Alter als neuer Aufbruch (*n* = 298; in %)
ärgert mich	38,3	50,5	27,5
bedrückt mich	73,9	39,6	26,2
bringt mich zum Schmunzeln	1,3	40,3	57,3
erregt mein Mitleid	82,5	18,8	8,0
erstaunt mich	23,9	48,2	34,3
macht mir Angst	66,3	25,4	16,8
macht mir Mut	16,8	15,8	70,1
schockiert mich	69,9	27,7	9,4
stimmt mich fröhlich	3,2	5,9	61,1
weckt Schuldgefühle in mir	14,6	11,2	34,3
löst keine Gefühle bei mir aus	25,9	54,1	31,9

Frame 1 wird von den Befragten als glaubwürdig, sachlich und überzeugend empfunden (Tab. 4). Auch Frame 3 erhält einen hohen Zustimmungswert in puncto Glaubwürdigkeit, aber auch Anschaulichkeit. Demgegenüber wird Frame 2 von den Befragten als eindeutig unglaubwürdig, übertrieben und provokant erachtet.

**Table 4:** 

Der Text ist …	1: Alter als Niedergang (*n* = 309; in %)	2: Alter als (Über)Macht (*n* = 303; in %)	3: Alter als neuer Aufbruch (*n* = 298; in %)
anschaulich	82,8	51,8	85,9
ausgewogen	50,7	24,8	64,8
glaubwürdig	75,2	40,3	68,5
interessant	65,4	58,1	88,3
provokant	37,7	84,2	41,9
sachlich	64,8	27,7	61,4
übertrieben	38,2	70,0	46,7
überzeugend	73,5	31,7	69,6

Nach dem Stimuluseinsatz wurden die Probanden gefragt, wie sie das Verhältnis zwischen Alt und Jung einschätzen (Tab. 5). Hierbei urteilen Befragte, die den zweiten Frame erhielten, merklich anders als die Befragten der beiden anderen Gruppen.

**Table 5:** 

Ältere Menschen …	1: Alter als Niedergang (*n* = 309)	2: Alter als (Über)Macht (*n* = 303)	3: Alter als neuer Aufbruch (*n* = 298)
kümmern sich zu wenig um die Zukunft der Jüngeren	2,8	3,3	2,8
bekommen vom Staat mehr, als ihnen zusteht	3,1	3,8	3,2
werden mit ihren Bedürfnissen von den Jüngeren vernachlässigt	2,7	2,1	2,8
haben das aufgebaut, wovon die Jüngeren heute zehren	2,4	1,7	2,5

Niedrige Werte: hohe Zustimmung, vierstufige Likert-Skala

Im Vorher-nachher-Vergleich fällt auf, dass das Fremdbild für die 6 wiederholten Items meist deutlich stärkere Veränderungen zeigt als das Selbstbild (Tab. 6). Zudem klaffen Selbst- und Fremdbildindikatoren auseinander. Im Fall von Frame 1 verbessert sich das Selbstbild moderat, das Fremdbild hingegen verschlechtert sich deutlich. Der exakt entgegengesetzte Trend lässt sich für die dritte Gruppe beobachten. Bezeichnenderweise verbessert sich in diesem Fall das Fremdbild erheblich, wohingegen das Selbstbild bei 2 Items Einbußen erleidet. Teilweise sind mittlere bis starke Effektstärken zu beobachten. Für Frame 2 sind keine Veränderungen im Ausmaß der anderen Gruppen festzustellen.

**Table 6:** 

Älterwerden/Alter bedeutet (für mich) …		1: Alter als Niedergang (*n* = 309)		2: Alter als (Über)Macht (*n* = 303)		3: Alter als neuer Aufbruch (*n* = 298)		
immer mehr Angst haben	*Selbstbild* vorher/nachher		3,0/3,3* Standardabw. = 0,979 Standardfehler = 0,056 T = −5,75 df = 308 Sig. (2-seitig) = 0,000 Cohen’s d = 0,33		2,9/2,9 Standardabw. = 0,276 Standardfehler = 0,016 T = −0,208 df = 302 Sig. (2-seitig) = 0,835 Cohen’s d = 0,03		2,7/2,4* Standardabw. = 0,909 Standardfehler = 0,053 T = 4,97; df = 297 Sig. (2-seitig) = 0,000 Cohen’s d = 0,29	
	*Fremdbild* vorher/nachher		2,3/1,8* Standardabw. = 0,705 Standardfehler = 0,04 T = 12,02 df = 308 Sig. (2-seitig) = 0,000 Cohen’s d = 0,68		2,5/2,5 Standardabw. = 0,191 Standardfehler = 0,011 T = −0,301 df = 302 Sig. (2-seitig) = 0,764 Cohen’s d = 0,01		2,2/2,9* Standardabw. = 0,839 Standardfehler = 0,049 T = −14,37; df = 297 Sig. (2-seitig) = 0,000 Cohen’s d = 0,83	
leben weiterhin selbst bestimmen	*Selbstbild* vorher/nachher		1,8/1,5* Standardabw. = 0,824 Standardfehler = 0,047 T = 6,14 df = 308 Sig. (2-seitig) = 0,000 Cohen’s d = 0,35		1,7/1,7 Standardabw. = 0,181 Standardfehler = 0,01 T = −1,26 df = 302 Sig. (2-seitig) = 0,206 Cohen’s d = 0,05		1,6/1,9* Standardabw. = 0,711 Standardfehler = 0,041 T = −7,65; df = 297 Sig. (2-seitig) = 0,000 Cohen’s d = 0,45	
	*Fremdbild* vorher/nachher		2,3/2,8 * Standardabw. = 0,573 Standardfehler = 0,033 T = −14,79 df = 308 Sig. (2-seitig) = 0,000 Cohen’s d = 0,86		2,2/2,2 Standardabw. = 0,73 Standardfehler = 0,042 T = −0,236 df = 302 Sig. (2-seitig) = 0,814 Cohen’s d = 0,01		2,3/1,8 * Standardabw. = 0,906 Standardfehler = 0,052 T = 8,57; df = 297 Sig. (2-seitig) = 0,000 Cohen’s d = 0,5	
nur noch für sich selbst interessieren	*Selbstbild* vorher/nachher		3,5/3,6 Standardabw. = 0,731 Standardfehler = 0,042 T = −1,56 df = 308 Sig. (2-seitig) = 0,121 Cohen’s d = 0,08		3,6/3,5 Standardabw. = 0,412 Standardfehler = 0,024 T = 2,23 df = 302 Sig. (2-seitig) = 0,026 Cohen’s d = 0,12		3,2/3,2 Standardabw. = 0,839 Standardfehler = 0,049 T = −0,345; df = 297 Sig. (2-seitig) = 0,73 Cohen’s d = 0,02	
	Fremdbild vorher/nachher		2,7/2,4 * Standardabw. = 0,638 Standardfehler = 0,036 T = 8,38 df = 308 Sig. (2-seitig) = 0,000 Cohen’s d = 0,47		3,1/2,6 * Standardabw. = 0,712 Standardfehler = 0,041 T = 11,13 df = 302 Sig. (2-seitig) = 0,000 Cohen’s d = 0,63		2,6/2,9 * Standardabw. = 0,908 Standardfehler = 0,053 T = −6,06; df = 297 Sig. (2-seitig) = 0,000 Cohen’s d = 0,35	

Niedrige Werte: hohe Zustimmung, vierstufige Likert-Skala

*Mittelwertdifferenz zwischen vorher und nachher auf dem Niveau *p* < 0,001

Vollständige Formulierung der Vorher-nachher-Items für das Selbstbild: *Älterwerden bedeutet für mich persönlich … 1) dass ich immer mehr Angst habe*; *2) dass ich mein Leben weiterhin selbst bestimmen kann*; *3) dass ich mich nur noch für mich selbst interessiere*

Vollständige Formulierung der Vorher-nachher-Items für das Fremdbild: *Alter bedeutet … 1) dass einem immer mehr Dinge Angst machen; 2) dass man sein Leben weiterhin selbst bestimmen kann*; *3) dass man sich nur noch für sich selbst interessiert*

## Diskussion

Die Studie hat sich mit der bislang vernachlässigten Frage nach den Wirkungen medienvermittelter Altersdarstellungen befasst. Die Zielgruppe stellten dabei ältere Personen dar. Auffällig ist, dass im Vorher-nachher-Vergleich des Stimuluseinsatzes Altersfremdbildindikatoren und -selbstbildindikatoren auseinander klaffen. So verbessert sich bei Vorlage der negativen Altersdarstellung das Selbstbild moderat, das Fremdbild hingegen verschlechtert sich deutlich. Umgekehrt verhält es sich bei der positiven Altersdarstellung, wo das Fremdbild eine Aufwertung erfährt, während sich das Selbstbild verschlechtert. Die formulierte Hypothese ist somit widerlegt worden, da die Veränderungsrichtungen von Altersselbstbild und -fremdbild sich unterscheiden.

Im Lichte der Ergebnisse lässt sich die Theorie sozialer Vergleichsprozesse heranziehen [6]. Ihr zufolge geben Medien Rezipienten Vergleichsmaßstäbe an die Hand. Für den Frame „Alter als Niedergang“ kann vermutet werden, dass die Probanden einen sozialen Abwärtsvergleich vornehmen. Konfrontiert mit der Dramatik des Altseins, kommt es zu einer Aufwertung des Selbstbilds, indem den Versuchspersonen bewusst wird, wie gut es ihnen im Vergleich zu den gebotenen älteren Menschen geht. Im Fall des Frame „Alter als neuer Aufbruch“ sehen sich die Probanden einem sozialen Aufwärtsvergleich ausgesetzt. Da die präsentierten Best agers durch ihre Aktivität und Fitness traditionelle Altersvorstellungen ad absurdum führen, reagiert ein Teil der Probanden eher mit Selbstzweifeln. Verschiedene Autoren verweisen auf das latente Diskriminierungspotenzial, das positiv verklärten Altersdarstellungen innewohnt [26].

Ein weiterer Aspekt ist, dass abhängig vom Frame-Stimulus die Beurteilung des Generationenverhältnisses unterschiedlich ausfallen kann. So kann im Fall von Frame 2 vermutet werden, dass die medial inszenierte Schürung eines von älteren Menschen ausgehenden Generationenkonflikts eine Gegenreaktion aufseiten der älteren Rezipienten provoziert, die sich auf erbrachte Leistungen und wohlfahrtsstaatliche Ansprüche, aber auch auf ein vermeintlich vernachlässigendes Verhalten seitens jüngerer Menschen bezieht.

Die Ergebnisse liegen insgesamt nah an jenen der ursprünglichen Studie und bestätigen damit mit einer deutlich größeren Stichprobe alle wesentlichen Befunde, die einstellungsverändernde Effekte medialer Altersdarstellungen vermuten lassen^4^. Wie auch in der ursprünglichen Arbeit waren keine nennenswerten Unterschiede zwischen Alters‑, Bildungs- und Geschlechtergruppen oder ein Einfluss intervenierender Variablen feststellbar, was aus Sicht der Verfasser für übergreifende Wirksamkeitspotenziale medienvermittelter Darstellungsmuster des höheren Lebensalters spricht [10, 12, 29].

Die Studie ist anschlussfähig an die Arbeit von Pinquart [19], der ältere Menschen mit negativen Altersstereotypen konfrontierte. Auch er stellte fest, dass sich in der Experimentalgruppe, die negative Informationen über Kompetenzen im Alter erhielt, generelle Einschätzungen über ältere Menschen verschlechterten, während sich die Selbsteinschätzungen verbesserten. Nicht unerwähnt bleiben soll die Arbeit von Mares und Cantor [13], die herausfanden, dass ältere Menschen beim Fernsehen gezielt soziale Auf- und Abwärtsvergleiche mit älteren TV-Figuren herstellen. Vor diesem Hintergrund fällt die Wirkung medialer Altersbilder in Abhängigkeit von individuellen Merkmalen, Motivationen und Stimmungen der Rezipienten unterschiedlich aus [23].

### Limitationen

Die Studie sollte als erster, explorierender Zugang zu einem weitgehend unerforschten Gebiet verstanden werden. Trotz der großen Stichprobe und der prägnanten, nun erneut bestätigten Ergebnisse weist sie verschiedene Schwachpunkte auf:

- Es handelte sich um textuelle Frames.
- Die Studie hat keine „natürlich“ auftretenden Altersdarstellungen rezipieren lassen.
- Die gemessenen Veränderungen des Altersfremdbilds und -selbstbilds sind kurzfristige Effekte. Es stellt sich die Frage, wie sich mediale Altersdarstellungen im längerfristigen Rezeptionsprozess unter Alltagsbedingungen auswirken.
- Die Veränderung von Altesfremdbild und -selbstbild wurde an einer sehr kleinen Zahl von Indikatoren gemessen.
- Zur Messung der Veränderung von Altersfremdbild und -selbstbild wurden Mittelwertvergleiche auf Gruppenebene durchgeführt und keine Vorher-nachher-Messungen auf Individualebene.
- Es wurde nicht gemessen, wie beeinflussbar ein Altersfremdbild bzw. -selbstbild relativ zu subjektiv bestehenden Voreinstellungen in Bezug auf Alter(n) ist.
- Im Vorfeld hat keine Power-Analyse zur Bestimmung eines angemessenen Stichprobenumfangs stattgefunden. Aufgrund dessen kann es sein, dass die Stichprobe zu groß geraten ist und die gemessenen Effekte überschätzt werden.

## Ausblick

Medienvermittelten Altersbildern kommt große Bedeutung zu, da sie gesellschaftliche Vorstellungen vom höheren Lebensalter maßgeblich mitprägen. Traditionell sind Altersbilder in Medienerzeugnissen inhaltsanalytisch untersucht und dann Effekte antizipiert worden. Die Ergebnisse zeigen indes, dass mediale Altersdarstellungen eine ambivalentere Wirkung haben können, als ihr inhaltlicher Gehalt vermuten lässt. So scheinen negative Altersdarstellungen mitunter dazu zu führen, dass sich ältere Personen in ihrer vermeintlichen Abgrenzung von der Kategorie „alt“ leichter bestätigt fühlen („Alt sind immer nur die anderen“), wohingegen (über)positive Altersrepräsentationen diese Abgrenzung u. U. erschweren.

Insgesamt lassen sich die Befunde mit der Theorie sozialer Vergleichsprozesse vereinbaren. Diese basiert auf der Annahme, dass Mediendarstellungen zu abwärts- und aufwärtsgerichteten Vergleichen genutzt werden können. Auch, wenn hier letztlich nur kurzfristige Effekte festgestellt werden konnten, sind die Ergebnisse als Hinweis darauf zu werten, dass nicht vorschnell von inhaltsanalytischen Feststellungen auf mögliche Wirkungen geschlossen werden sollte. Dies würde der Komplexität möglicher Einflusspotenziale durch mediale Altersbilder nicht gerecht werden, derer sich die weitere empirische Forschung annehmen sollte. Dabei sollte in Zukunft gerade die komplexe Beziehung von Altersfremdbildern und -selbstbildern bei der Wirkung von Altersdarstellungen beachtet werden.

## Fazit für die Praxis

- In Zeiten demografischer Veränderungen, zurückgehender Generationenkontakte und der gesellschaftlichen Mediatisierung haben mediale Altersbilder großen Einfluss auf individuelle Altersvorstellungen und den Altersdiskurs.
- Bislang liegen nahezu keine empirischen Befunde darüber vor, welche (einstellungsverändernden) Effekte medienvermittelte (Re)Präsentationen des Alters haben können. Denkbar sind unterschiedliche Wirkungspotenziale, die das Altersfremdbild und -selbstbild beeinflussen.
- Die Ergebnisse zeigen, dass sich mediale Altersdarstellungen nicht so auswirken, wie sie inhaltlich beschaffen sind, sondern sogar gegenteilige Effekte haben können, wenn sie etwa für soziale Vergleichsprozesse genutzt werden.
- Eine genauere Erforschung medienvermittelter Altersdarstellungen erscheint angesichts ihrer komplexen Wirkmechanismen angeraten. Besonders gilt dies mit Blick auf die Interaktion von Altersfremdbild und -selbstbild.

## Caption Electronic Supplementary Material






## Footnotes

1 Die Bezeichnung „quasiexperimentell“ hängt damit zusammen, dass nicht alle Eigenschaften erfüllt sind, die für ein vollständiges soziales Experiment erforderlich wären. So lässt sich eine zentrale Variable – etwaige bereits vorhandene Altersvorstellungen – nicht vom Versuchsleiter kontrollieren, da altersbezogene Dispositionen schon lange vor der Durchführung in den Köpfen der Probanden präsent sind und auf mannigfache individuelle Eindrücke, Gefühle, Meinungen sowie Erfahrungen mit dem höheren Lebensalter zurückgehen. Zudem erfolgte die Aufteilung der Probanden auf die Experimentalgruppen nicht nach Randomisierungskriterien, sondern forschungspragmatisch in größeren Klumpen (in der Regel ein bestimmter Stimulus pro befragter Seniorengruppe).

2 Besagte Indikatoren wurden dem deutschen Alterssurvey entnommen [17].

3 Entsprechend der Altersstruktur der älteren Bevölkerung [25] sollte jede Gruppe in etwa folgende Zusammensetzung haben: ca. 48 % 60 bis 70 Jahre, ca. 43 % 71 bis 85 Jahre, ca. 9 % 86+ Jahre. Das Verhältnis zwischen Frauen und Männern jeder Altersgruppe sollte ausgeglichen sein.

4 Die 2013 geführten fokussierten Interviews mit älteren Menschen, die mit den 3 Frame-Prototypen konfrontiert wurden, bekräftigen die in der Befragung erhaltenen Ergebnisse [29].
