# Supplementary material for: What effects do media-mediated images of old age have on older people?—Results of a reception study
Source: Z Gerontol Geriatr. 2020 Jun 30;54(7):676–84. [Article in German] doi: 10.1007/s00391-020-01745-y (PMC8551094; doi:10.1007/s00391-020-01745-y)
Supplement: Supplementary file 1 [file 391_2020_1745_MOESM1_ESM.doc]

**Frame-Identifikation: Kodierleitfaden (zentrale Kategorien)**

| **Framedefinierende**  **Kategorie/Definition** | **Kodierregeln/Ausprägungen** |
| --- | --- |
| **Thema**  Welches Thema behandelt der Beitrag? |  |
| **Akteursstatus**  Kommen ältere Menschen im Beitrag selbst zu Wort und/oder beeinflussen sie das Geschehen in irgendeiner Weise aktiv? | - Aktiv - Passiv - Nicht erkennbar |
| **Zahlenmäßiges Auftreten**  In welcher Zahl/Konstellation treten ältere Menschen im Beitrag auf? | - Einzelperson - Paar - Gruppe - Masse - Nicht erkennbar |
| **Umfeld**  In welcher sozialen Umgebung sind ältere Menschen im Beitrag anzutreffen? | - Berufliches Umfeld - Häusliches/familiäres Umfeld - Freizeitumfeld - Pflegeheim/Krankenhaus - Öffentliches Umfeld - Sonstiges Umfeld - Nicht erkennbar |
| **Geschlecht**  Welchem Geschlecht sind ältere Menschen im Beitrag zuzuordnen? | - Männlich - Weiblich - Beide - Nicht erkennbar |
| **Schichtzugehörigkeit**  Welche materielle Lebenslage älterer Menschen wird im Beitrag dargeboten? | Codiert wird die materielle Lebenslage älterer Menschen. Die Zuordnung einzelner Personen in eine soziale Schicht erfolgt über den Beruf oder nachfolgende Erkennungsmerkmale:   - Oberschicht (sehr gutes Auskommen, Repräsentanten aus Politik, Wirtschaft, Verbänden, Kirche etc.) - Mittelschicht (gutes bis hinreichendes Auskommen, höherer Schulabschluss und/oder Studium, oftmals berufliche Selbstständigkeit, Entscheidungsbefugnis im Arbeitsvollzug, häufiger Besitz hochwertiger Konsumgüter, ausgeprägtes politisches und Kulturinteresse, individuelle Lebensgestaltung, elaborierte Sprachmöglichkeiten etc.) - Unterschicht (sozioökonomisch benachteiligt, überwiegend Volksschulabschluss, abhängige Beschäftigung, geringe Entscheidungskompetenz, geringer Konsum, schwach ausgeprägtes Kulturinteresse, starker Bezug zur eigenen Schicht, einfache Sprache, für Mitmenschen wenig bedeutend etc.) - Nicht erkennbar |
| **Beziehung Alt-Jung**  Wie ist das Verhältnis zwischen älteren und jüngeren Menschen im Beitrag beschaffen? | - Abhängigkeitsverhältnis - Harmonisches Verhältnis - Neutrales bis gleichgültiges Verhältnis - Spannungsreiches Verhältnis - Nicht erkennbar |
| **Beziehung Alt-Alt**  Wie ist das Verhältnis zwischen älteren Menschen im Beitrag beschaffen? | - Harmonisches Verhältnis - Solidarisches Verhältnis - Distanziertes Verhältnis - Nicht erkennbar |
| **Zugeschriebene Rolle**  Welche zentrale Rolle nehmen ältere Menschen innerhalb des Beitrags ein? Welche expliziten oder impliziten Eigenschaften beinhaltet diese Rolle? | Codiert wird die Rolle, die älteren Menschen zugeschrieben wird:   - Golden Ager (v.a. aktiv, fit, gesundheitsbewusst, gut informiert, lebensfroh, produktiv, selbstständig, unabhängig, zukunftsorientiert) - Perfekte Großeltern (v.a. familienorientiert, fürsorglich, hilfsbereit, liebenswert, liebevoll, traditionell, sympathisch, unterstützend, vertrauenswürdig) - Elder Statesman/Altersikone (v.a. beruflich charakterisiert, erfahren, erfolgreich, intelligent, reich, resolut, stilsicher, stolz, unerschöpflich, willensstark) - Exzentrisches Alter (v.a. altmodisch, bieder, arrogant, atypisch, eitel, lächerlich, schrullig, selbstverliebt, skurril, verschroben) - Eingeschränktes und gehemmtes Alter (v.a. ängstlich, armselig, besorgt, depressiv, einsam, entkräftet, frustriert, gebrechlich, hilfsbedürftig/hilflos, inkompetent, krank, niedergeschlagen, senil, unselbstständig, verzweifelt) - Griesgrämiges Alter (v.a. fordernd, konfrontativ, schmarotzend, selbstbezogen, übellaunig, unflexibel, unzufrieden, verbittert, verstockt, zänkisch) |
| **Bewertungstendenz**  Welche Bewertungstendenz im Hinblick auf Alter bzw. ältere Menschen ist dem Beitrag zu entnehmen? | Codiert wird der Grundtenor des Artikels in Bezug auf ältere Menschen und/oder Alter:   - Eindeutig positiv - Eher positiv - Ambivalent - Eher negativ - Eindeutig negativ - Nicht erkennbar |
