# Supplementary material for: What effects do media-mediated images of old age have on older people?—Results of a reception study
Source: Z Gerontol Geriatr. 2020 Jun 30;54(7):676–84. [Article in German] doi: 10.1007/s00391-020-01745-y (PMC8551094; doi:10.1007/s00391-020-01745-y)
Supplement: Supplementary file 2 [file 391_2020_1745_MOESM2_ESM.doc]

1. **Fangen wir mit der folgenden Frage an: Es gibt ja Menschen, die morgens munterer sind, andere kommen erst abends richtig in Schwung. Wie ist das bei Ihnen?**

**
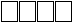
**

**Ich bin morgens munterer.**

**
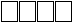
**

**Ich komme erst abends richtig in Schwung.**

**
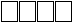
**

**Unentschieden/teils, teils.**

1. **Was schätzen Sie: Wie häufig nutzen Sie die folgenden Medien?**

|  | **Nie** | **Selten** | **Gelegentlich** | **Oft** | **Sehr häufig** |
| --- | --- | --- | --- | --- | --- |
| **Fernsehen** |  |  |  |  |  |
| **Zeitung/**  **Zeitschrift** |  |  |  |  |  |
| **Radio** |  |  |  |  |  |
| **Internet** |  |  |  |  |  |
| **Andere, und zwar:** | | | | | |

1. **Wenn Sie einmal an das Angebot an Zeitungen und Zeitschriften denken: Bitte kreuzen Sie alle aufgelisteten Medienangebote an, die Sie regelmäßig nutzen.**


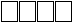
**Tageszeitung**

(z.B. Regionalzeitung, *Süddeutsche Zeitung*, *Frankfurter Allgemeine Zeitung*)


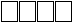
**Wochenzeitung**

(z.B. *Die Zeit*)


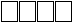
**Sonntagszeitung**

(z.B. *Bild am Sonntag*, *Frankfurter Allgemeine Sonntagszeitung*, *Welt am Sonntag*)


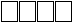
**Magazine**

(z. B. *Focus*, *Spiegel*, *Stern*)

**
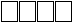
**

**Illustrierte**

(z.B. *Bunte*, *Gala*, *Das Goldene Blatt*, *Super Illu*)


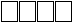
**Kundenzeitschrift**

(z.B. *Apotheken Umschau*)


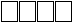


**andere Zeitung/Zeitschrift, und zwar: ________________________**

1. **Es gibt verschiedene Gründe, weshalb man Medien (Fernsehen, Zeitung/Zeitschrift, Radio, Internet) nutzt. Bitte kreuzen Sie an, inwieweit die folgenden Gründe auf Sie zutreffen.**

| **Ich nutze Medien,** | **Trifft voll und ganz zu** | **Trifft eher zu** | **Trifft eher nicht zu** | **Trifft überhaupt nicht zu** |
| --- | --- | --- | --- | --- |
| **…damit ich mitreden kann.** |  |  |  |  |
| **…um mich zu unterhalten.** |  |  |  |  |
| **…um zu sehen, wie andere Menschen so leben.** |  |  |  |  |
| **…weil es mir hilft, meinen Alltag zu strukturieren.** |  |  |  |  |
| **…weil ich dadurch erfahre, dass andere ähnliche Probleme haben.** |  |  |  |  |
| **…weil ich damit den Alltag vergessen kann.** |  |  |  |  |
| **…weil ich Denkanstöße bekomme.** |  |  |  |  |
| **…weil ich mich dann nicht allein fühle.** |  |  |  |  |
| **…weil ich mich informieren möchte.** |  |  |  |  |
| **…weil ich Nützliches für den Alltag erfahre.** |  |  |  |  |

1. **Wenn Sie an Journalismus denken: Glauben Sie, dass Journalisten ehrlich mit ihrem Publikum umgehen?**

**
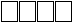
**

**Ja, Journalisten gehen ehrlich mit ihrem Publikum um.**

**
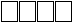
**

**Nein, Journalisten gehen nicht ehrlich mit ihrem Publikum um.**

**
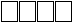
**

**Unentschieden/teils, teils.**

1. **Ab welchem Alter würden Sie jemanden als ‚alt‘ bezeichnen?**

**____** Jahre

1. **Wenn Sie an Ihr eigenes Älterwerden denken: Inwieweit treffen die folgenden Aussagen auf Sie zu? Bitte stufen Sie ab.**

| **Älterwerden bedeutet für mich persönlich, dass…** | **Trifft voll und ganz zu** | **Trifft eher zu** | **Trifft eher nicht zu** | **Trifft überhaupt nicht zu** |
| --- | --- | --- | --- | --- |
| **...ich mich mit der Zeit immer häufiger langweile.** |  |  |  |  |
| **…ich genauer weiß, was ich will.** |  |  |  |  |
| **…mir immer mehr Dinge Angst machen.** |  |  |  |  |
| **…ich vielen Dingen gegenüber gelassener werde.** |  |  |  |  |
| **…ich mich nur noch für mich selbst interessiere.** |  |  |  |  |
| **…ich mein Leben weiterhin selbst bestimmen kann.** |  |  |  |  |
| **…ich weniger respektiert werde.** |  |  |  |  |

1. **Haben Sie es gern, wenn ein Garten mit Gartenzwergen geschmückt ist?**

**
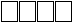
**

**Ja, ich habe es gern.**

**
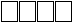
**

**Nein, ich habe es nicht gern.**

**
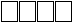
**

**Unentschieden/es kommt darauf an.**

1. **Gibt es einen Fernsehfilm oder eine Fernsehserie, die Ihnen besonders gut gefällt?**

____________________________________

1. **Nun folgen einige Aussagen zum Alter allgemein. Bitte gehen Sie bei der Beantwortung nicht von Ihrem eigenen Älterwerden aus. Inwieweit treffen die folgenden Aussagen Ihrer Meinung nach auf Alter zu? Bitte stufen Sie ab.**

| **Alter bedeutet, dass…** | **Trifft voll und ganz zu** | **Trifft eher zu** | **Trifft eher nicht zu** | **Trifft überhaupt nicht zu** |
| --- | --- | --- | --- | --- |
| **…man sich häufiger einsam fühlt.** |  |  |  |  |
| **…man bewusster lebt als in jüngeren Jahren.** |  |  |  |  |
| **…man nicht mehr so recht gebraucht wird.** |  |  |  |  |
| **…man sein Leben weiterhin selbst bestimmen kann.** |  |  |  |  |
| **…einem immer mehr Dinge Angst machen.** |  |  |  |  |
| **…man genauso glücklich ist, wie man es in jüngeren Jahren war.** |  |  |  |  |
| **…man sich nur noch für sich selbst interessiert.** |  |  |  |  |

**Der nachfolgende Artikel wurde vor kurzem in einem bekannten Nachrichtenmagazin veröffentlicht. Bitte lesen Sie ihn sich in Ruhe durch und blättern Sie erst danach weiter.**

(Stimulustext 1 – Frame 1: *Alter als menschlicher Niedergang*)

**Wo auch die Würde stirbt**

**Der letzte Weg ist lang und schäbig. Hunderttausende von Alten dämmern in deutschen Heimen und Krankenhäusern ihrem Ende entgegen – gut verwaltet, aber körperlich und seelisch vernachlässigt. Resignierte Pfleger und Experten klagen: Selbst Tiere werden besser geschützt.**

Je älter man wird, desto größer ist die Wahrscheinlichkeit, in einem Alten- oder Pflegeheim zu sterben. Nur dauert in Zeiten der Intensivmedizin das Sterben nicht mehr wenige Wochen wie früher. Die Folge: Viele Monate, manchmal Jahre des Dahinvegetierens. Man lässt den Körper nicht den Weg gehen, den der Geist oft schon genommen hat, wenn Vergesslichkeit zur Demenz geworden ist. Dann bleibt oft nur das Pflegeheim.

Unterernährung, Austrocknung, Gesundheitsschädigungen durch Beruhigungsmittel, großvolumige Inkontinenzeinlagen, die gegen den Willen der Pflegebedürftigen eingesetzt und unangemessen lang nicht gewechselt werden, Verletzungen der persönlichen Integrität und Würde bis hin zur Freiheitsberaubung sind die Regel. Pflegeexperte Claus Fussek flüchtet sich in schwarzen Humor: „Was ist der Unterschied zwischen Altöl und alten Menschen? Bei der Öl-Entsorgung sind gewisse Standards einzuhalten.“ Einmal hat er den Chef eines Caritasverbandes gefragt, ob er folgende Minimalstandards in seinen Heimen garantieren könne: „Zu essen und zu trinken im Kau- und Schlucktempo, waschen, anziehen, Gebiss rein, zur Toilette so oft, wie gewollt, einmal am Tag an die frische Luft, beim Sterben die Hand halten.“ „Leider nein“, hat der ehrliche Mann geantwortet.

Hinzu kommt: Krankenhäuser entlassen Pflegefälle rigoros, oft in erbärmlichem Zustand. Sie werden ins Pflegeheim abgeschoben. Dort sammelt sich eine Klientel, die immer älter und kränker wird.

Krass, aber nicht ungewöhnlich ist das Beispiel des Münchners Michael Nunhofer. Er musste seine 87-jährige Mutter Maria aus dem Krankenhaus nach Hause holen, obwohl an ihrem Rücken noch eine riesige Wunde klaffte. Die war groß wie eine Hand und so tief, dass die Wirbelsäule durchschimmerte: ein eitriges Druckgeschwür, das die alte Dame sich durch Pflegefehler erst geholt hatte. Einen Monat später war sie tot.

Je jünger die Todkranken sind, desto wahrscheinlicher kommt das Ende im Krankenhaus. Zwischen 60 und 80 Jahren stirbt mehr als jeder Zweite in einer Klinik. Bei den noch Älteren sinkt der Anteil auf ein Viertel. Erfahrene Heimleiter vermeiden, dass ihre sterbenden Pfleglinge noch in letzter Sekunde ins Krankenhaus kommen, „weil sie dort nur an Schläuche gehängt werden“.

Angesichts der 100.000 Menschen, die heute schon an Ernährungsschläuchen hängen – Tendenz steigend –, hat Contz Hilber, Chefarzt des Kreiskrankenhauses Mühldorf, eine Horrorvision: „Irgendwann gibt es Stationen mit Nahrungsleitungen wie im Kuhstall. Abzweige gehen in jedes Bett, und im Keller steuert alles ein Computer.“

(Stimulustext 2 – Frame 2: *Alter als (Über)Macht*)

**Die graue Macht**

**Sie sind alt, sie sind kampflustig, und sie sind viele. 20 Millionen Wähler zählen 60 Jahre und mehr. Ohne sie ist keine Wahl zu gewinnen. Um sie milde zu stimmen, hat die Regierung mitten in der Krise die Renten hochgesetzt. Szenen aus einem Land auf dem Weg zur Rentner-Demokratie.**

Eine graue Macht, die durch ihre schiere Masse unter Politikern Angst verbreitet – und so über die jüngeren Generationen herrscht. Deutschland ist längst auf dem Weg in eine Rentner-Demokratie. Weltwirtschaftskrise? Haushaltslöcher? Alles egal. Egal, dass die Rentenkasse eigentlich schon heute pleite ist.

Dass die jüngeren Arbeitnehmer nicht mehr auf eine auskömmliche Rente hoffen können, sondern selbst vorsorgen müssen. Alles egal. Die Jüngeren mögen die Wirtschaft am Laufen halten, das Bruttosozialprodukt und damit auch die Renten erarbeiten, aber die wichtigste Zielgruppe im Kampf um die Macht sind längst die Wähler jenseits der 60. Die Generation Silber. S-Klasse. Best-Ager. Die wissen das auch nur zu gut.

„Ohne die Älteren, ob man es begrüßt oder nicht, ist auf Dauer keine Wahl mehr zu gewinnen“, sagt Otto Wulff, Chef der Altenorganisation Senioren-Union. „Die Älteren spüren das und werden sich mehr und mehr ihrer Macht bewusst.“ Mit unverhohlenem Vergnügen beobachtet er, mit welch wachsendem Feuereifer sich die Parteistrategen Gedanken um seine Altersgenossen machen. Mit 20 Millionen Stimmen stellen die Senioren schon heute ein Drittel aller Wähler. Eine einfache Formel: Keine Alten – keine Macht.

Trotz ihrer vielen Privilegien fühlen sich viele Rentner ungerecht behandelt. Dass Altersarmut fast verschwunden ist, dass heute alleinerziehende Mütter und ihre Kinder arm dran sind, dass es noch nie einer Seniorengeneration so gut ging – davon wollen viele nichts wissen. „Keine Partei kann es sich leisten, die Älteren zu verärgern“, sagt Manfred Güllner vom Meinungsforschungsinstitut Forsa.

Dabei zeigt sich der Einfluss der Alten weniger in konkreten Versprechen. Die Macht der Senioren ist diffuser. Sie schmerzt in den Köpfen der Regierenden und lässt sie in Wahlkampfzeiten vorsichtig durch mögliche Minenfelder lavieren. Nur ja keine unangenehmen Wahrheiten! Der demografische Wandel? Nicht mehr als eine spannende Herausforderung! Die Rente? Sicher! Die Krise? Aber doch nicht für euch!

Jugendwahn war gestern. Bertold Bahner, 73, Vorsitzender der Liberalen Senioren, sitzt in seinem Wohnzimmer und spürt Aufwind für die Alten: „Wir stehen am Anfang einer neuen Ära. Die Gesellschaft und damit auch die Politik werden sich ändern. Den Alten gehört die Zukunft.“

(Stimulustext 3 – Frame 3: *Alter als neuer Aufbruch*)

**Golden Oldies: Generation Happy End**

**‚Das Alter‘ gibt es nicht mehr, und die mittleren Jahre dauern heute bis mindestens 70: Protokoll einer gesellschaftlichen Revolution.**

Wo sind die Dauerwellen? Weit und breit keine zu sehen. Die ‚Neuen Alten‘ werden in den nächsten Jahrzehnten die Gesellschaft prägen: Ihnen steht mehr Geld zur Verfügung als jüngeren Altersgruppen. Sie profitieren von einer seit 60 Jahren günstigen wirtschaftlichen Entwicklung ohne Kriege und extreme Geldentwertung.

Sie sind so fit wie keine Altengeneration vor ihnen, denn sie genießen die Segnungen der modernen Medizin, verhalten sich gesundheitsbewusst und legen Wert auf Mode. Vor allem viele Männer treiben noch intensiv Sport, manche laufen gar Marathon. Zudem sind in den vergangenen Jahrzehnten die Jobs mit schwerer körperlicher Arbeit stark zurückgegangen. Experten schätzen, dass der altersbedingte Abbau heute fünf bis zehn Jahre später beginnt als in früheren Generationen.

Sie sind geistig rege und mobil, wie es Alte noch nie waren. Knapp 20.000 Senioren sind Gasthörer an deutschen Universitäten. Zwei von drei 50- bis 59-Jährigen surfen im Netz, immerhin jeder Vierte über 60 tut das. Die meisten der älteren Deutschen fahren noch Auto, es wird verreist und konsumiert, bis die Kreditkarte qualmt.

Alter: Noch vor etwa 30 Jahren war das eine Zeit bescheidener Ansprüche und geringen Tatendrangs: kleine Wohnung, Kreuzworträtsel lösen, Blumen gießen, Heino im Radio hören, mit Dackel Waldi ums Quadrat gehen, ab und zu eine Kaffeefahrt mitmachen. Die Lebenswelt der heutigen Generation 55plus sieht dagegen oft so aus: Mechthild Gerdes, die Leiterin eines Wohnprojekts, ist so vielseitig interessiert und sozial engagiert, dass sie bisweilen in Terminnot gerät. Der Patentanwalt Utz Kador, 65, möchte bis 85 arbeiten und betreibt intensiv die verschiedensten Sportarten. Die 70-jährige Helga Herrmann lernt gerade vier Sprachen und schreibt ihren ersten Roman.

„Das Etikett ‚alt‘ wird einem von anderen aufgeklebt“, klagt Helga Herrmann, eine höchst aktive 70-Jährige. „Die Leute sagen: ,Kannst du das in deinem Alter noch machen? ‚In deinem Alter‘ – wenn ich das schon höre! Es gibt ja den Vorwurf, wir seien vom ‚Jugendwahn‘ befallen. Falsch, es ist Alterswahn, unter dem wir leiden, die Vorstellung, wir dürften dies und das nicht mehr tun!“

Die neuen Senioren, so schreiben die Autoren des Buches *Die Altersrevolution*, schaffen „eine völlig neue Vorstellung vom Leben im Alter, das nicht länger auf Gebrechen, Leid, Tristesse und Einsamkeit beschränkt ist, sondern Aktivität, Vitalität, Lebensfreude und Hoffnung für die Zukunft verheißt“.

**- BITTE BLÄTTERN SIE NICHT MEHR ZURÜCK -**

1. **Würden Sie sagen, Sie fühlen sich jetzt besser oder schlechter als vor dem Lesen des Textes?**

**
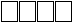
**

**Ich fühle mich besser.**

**
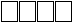
**

**Ich fühle mich schlechter.**

**
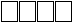
**

**Ich fühle mich genauso.**

1. **Wenn Sie an den Artikel zurückdenken: Wie werden ältere Menschen aus Ihrer Sicht darin dargestellt? Bitte stufen Sie ab.**

| **Im Artikel sind ältere Menschen…** | **Trifft voll und ganz zu** | **Trifft eher zu** | **Trifft eher nicht zu** | **Trifft überhaupt nicht zu** |
| --- | --- | --- | --- | --- |
| **…aufgeschlossen für Neues.** |  |  |  |  |
| **…eine treibende Macht in der Gesellschaft.** |  |  |  |  |
| **…ihrer Lage ausgeliefert.** |  |  |  |  |
| **…so aktiv und rege, dass sie ihr Alter gar nicht mitbekommen.** |  |  |  |  |
| **…um ihr letztes Fünkchen Würde gebracht.** |  |  |  |  |
| **…nur auf ihre Vorteile bedacht.** |  |  |  |  |

1. **Würden Sie sagen, Sie können sich im Großen und Ganzen mit den älteren Menschen im Artikel identifizieren?**

**
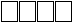
**

**Ja, im Großen und Ganzen kann ich mich mit ihnen identifizieren.**

**
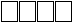
**

**Nein, diese älteren Menschen haben mit mir und meinem Leben nichts zu tun.**

**
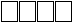
**

**Unentschieden/teils, teils.**

1. **Welche Gefühle hat der Text bei Ihnen ausgelöst? Bitte stufen Sie ab.**

| **Der Text…** | **Trifft voll und ganz zu** | **Trifft eher zu** | **Trifft eher nicht zu** | **Trifft überhaupt nicht zu** |
| --- | --- | --- | --- | --- |
| **…ärgert mich.** |  |  |  |  |
| **…bedrückt mich.** |  |  |  |  |
| **…bringt mich zum Schmunzeln.** |  |  |  |  |
| **…erregt mein Mitleid.** |  |  |  |  |
| **…erstaunt mich.** |  |  |  |  |
| **…macht mir Angst.** |  |  |  |  |
| **…macht mir Mut.** |  |  |  |  |
| **…schockiert mich.** |  |  |  |  |
| **…stimmt mich fröhlich.** |  |  |  |  |
| **…weckt Schuldgefühle in mir.** |  |  |  |  |
| **…löst keine Gefühle bei mir aus.** |  |  |  |  |

1. **Wenn Sie an den Artikel denken, den Sie eben gelesen haben (ohne zurückzublät-tern): Welchen Eindruck hatten Sie von dem Text? Bitte stufen Sie ab.**

| **Der Artikel war…** | **Trifft voll und ganz zu** | **Trifft eher zu** | **Trifft eher nicht zu** | **Trifft überhaupt nicht zu** |
| --- | --- | --- | --- | --- |
| **…anschaulich.** |  |  |  |  |
| **…ausgewogen.** |  |  |  |  |
| **…glaubwürdig.** |  |  |  |  |
| **…interessant.** |  |  |  |  |
| **…provokant.** |  |  |  |  |
| **…sachlich.** |  |  |  |  |
| **…übertrieben.** |  |  |  |  |
| **…überzeugend.** |  |  |  |  |

1. **Wenn Sie an Ihr eigenes Älterwerden denken: Inwieweit treffen die folgenden Aussagen auf Sie zu? Bitte stufen Sie ab.**

| **Älterwerden bedeutet für mich persönlich, dass…** | **Trifft voll und ganz zu** | **Trifft eher zu** | **Trifft eher nicht zu** | **Trifft überhaupt nicht zu** |
| --- | --- | --- | --- | --- |
| **…alles immer nur noch schlimmer wird.** |  |  |  |  |
| **…ich weiterhin in der Lage bin, neue Dinge zu lernen.** |  |  |  |  |
| **…ich mir selbst fremd werde, weil sich so vieles bei mir ändert.** |  |  |  |  |
| **…ich mein Leben weiterhin selbst bestimmen kann.** |  |  |  |  |
| **…mir immer mehr Dinge Angst machen.** |  |  |  |  |
| **…ich größeren Wert auf meine täglichen Gewohnheiten lege.** |  |  |  |  |
| **…ich mich nur noch für mich selbst interessiere.** |  |  |  |  |

1. **Die folgenden Aussagen befassen sich mit dem Verhältnis von Alt und Jung. Bitte stufen Sie ab, inwieweit die folgenden Aussagen Ihrer Meinung nach zutreffen.**

| **Ältere Menschen…** | **Trifft voll und ganz zu** | **Trifft eher zu** | **Trifft eher nicht zu** | **Trifft überhaupt nicht zu** |
| --- | --- | --- | --- | --- |
| **…kümmern sich zu wenig um die Zukunft der Jüngeren.** |  |  |  |  |
| **…bekommen vom Staat mehr als ihnen zusteht.** |  |  |  |  |
| **…werden mit ihren Bedürfnissen von den Jüngeren vernachlässigt.** |  |  |  |  |
| **…haben das aufgebaut, wovon die Jüngeren heute zehren.** |  |  |  |  |

1. **Nun folgen ein paar Aussagen darüber, wie ältere Menschen allgemein in den Medien dargestellt werden. Bitte kreuzen Sie nur eine Aussage an, und zwar die, die Ihrer Meinung nach am ehesten zutrifft.**

**
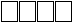
**

**Ältere Menschen werden in den Medien zu negativ dargestellt.**

**
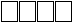
**

**Ältere Menschen werden in den Medien nicht anders als Jüngere dargestellt.**

**
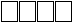
**

**Ältere Menschen werden in den Medien zu positiv dargestellt.**

**
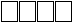
**

**Ältere Menschen werden von den Medien nicht mehr groß beachtet.**

**
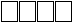
**

**Ältere Menschen werden von den Medien realistisch dargestellt.**

**
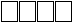
**

**Unentschieden/teils, teils.**

1. **Nun folgen einige Aussagen zum Alter allgemein. Bitte gehen Sie bei der Beantwortung nicht von Ihrem eigenen Älterwerden aus. Inwieweit treffen die folgenden Aussagen Ihrer Meinung nach auf Alter zu? Bitte stufen Sie ab.**

| **Alter bedeutet, dass…** | **Trifft voll und ganz zu** | **Trifft eher zu** | **Trifft eher nicht zu** | **Trifft überhaupt nicht zu** |
| --- | --- | --- | --- | --- |
| **…man häufiger unzufrieden mit sich selbst ist.** |  |  |  |  |
| **…man sein Leben weiterhin selbst bestimmen kann.** |  |  |  |  |
| **…man sich nur noch für sich selbst interessiert.** |  |  |  |  |
| **…man noch am Leben teilnimmt.** |  |  |  |  |
| **…einem immer mehr Dinge Angst machen.** |  |  |  |  |
| **…man ein Vorbild für jüngere Leute ist.** |  |  |  |  |
| **…man auf die Hilfe anderer angewiesen ist.** |  |  |  |  |

1. **Nun haben Sie es fast geschafft. Jetzt benötige ich noch ein paar Angaben zu Ihrer Person. Alle Angaben werden streng vertraulich und anonym behandelt.**

**Sie sind…**

**
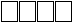
**

männlich


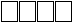


weiblich

**Sie haben folgende Staatsangehörigkeit…**

**
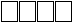
**

deutsch


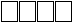


sonstige Staatsangehörigkeit, und zwar: **________________________**

**Wie viele Personen leben in Ihrem Haushalt, wenn Sie sich selbst mitzählen?**

**____** Person/en

**Wo wohnen Sie?**

**
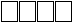
**

eigene Wohnung


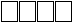


bei Angehörigen (Familie, Freunde)


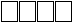


in einem Pflegeheim


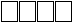


in einer Seniorenresidenz


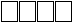


sonstige Wohnsituation, und zwar: **________________________**

**Welchen höchsten Bildungsabschluss besitzen Sie?**

**
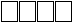
**

Volks-/Hauptschulabschluss


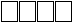


Mittlere Reife/Realschulabschluss


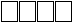


Abitur oder Fachabitur


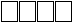


Fachhochschul- oder Hochschulabschluss


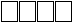


Abgeschlossene Berufsausbildung


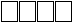


keinen Abschluss


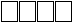


anderer Bildungsabschluss, und zwar: **________________________**

**Wie ist Ihr derzeitiger beruflicher Status?**

**
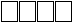
**

voll berufstätig


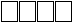


teilweise berufstätig


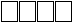


Rentner/in


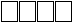


nicht berufstätig


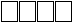


arbeitsuchend


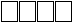


anderer beruflicher Status, und zwar: **________________________**

**Sind Sie ehrenamtlich tätig?**

**
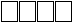
**

Ja, ich bin ehrenamtlich tätig.


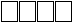


Nein, ich bin nicht ehrenamtlich tätig.

**Als was sind oder waren Sie hauptberuflich tätig?**

**
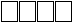
**

Arbeiter/Angestellter


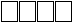


Beamter


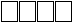


freiberuflich tätig


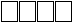


selbstständig tätig


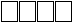


Hausfrau/Hausmann


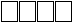


andere Berufstätigkeit, und zwar: **________________________**

**Wie häufig haben Sie Kontakt zu Mitgliedern Ihrer Familie?**

**
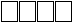
**

häufig


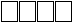


gelegentlich


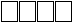


kaum


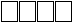


Meine Verwandten sind bereits verstorben.

**Wie häufig haben Sie Kontakt zu Freunden?**

**
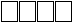
**

häufig


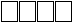


gelegentlich


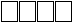


kaum


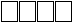


Meine Freunde sind bereits verstorben.

**Wie alt sind Sie?**

**____** Jahre

**Einmal abgesehen von Ihrem tatsächlichen Alter: Wenn Sie es in Jahren ausdrücken sollten, wie alt fühlen Sie sich?**

**____** Jahre

**Wenn Sie einmal Ihre Einkünfte überschlagen: Wie viel Geld steht Ihnen netto (also nach Abzug von Steuern und Sozialversicherung) monatlich zur Verfügung?**

**
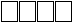
**

unter 500 Euro


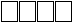


500 bis unter 1.000 Euro


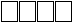


1.000 bis unter 2.000 Euro


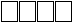


2.000 bis unter 3.000 Euro


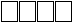


3.000 Euro und mehr

**Haben Sie noch Kritik oder Anmerkungen zum Fragebogen? Bitte tragen Sie diese hier ein.**

**Vielen Dank für Ihre Mitarbeit.**
